# The impact of cross-validation choices on pBCI classification metrics: lessons for transparent reporting

**DOI:** 10.3389/fnrgo.2025.1582724

**Published:** 2025-07-01

**Authors:** Felix Schroeder, Stephen Fairclough, Frederic Dehais, Matthew Richins

**Affiliations:** ^1^School of Psychology, Liverpool John Moores University, Liverpool, United Kingdom; ^2^Institut Supérieur de l'Aéronautique et de l'Espace (ISAE-SUPAERO), Université de Toulouse, Toulouse, France; ^3^Defence Science and Technology Laboratory, Salisbury, United Kingdom

**Keywords:** passive Brain-Computer Interfaces, pBCI, electroencephalography, EEG, cross-validation, non-stationarity, workload

## Abstract

Neuroadaptive technologies are a type of passive Brain-computer interface (pBCI) that aim to incorporate implicit user-state information into human-machine interactions by monitoring neurophysiological signals. Evaluating machine learning and signal processing approaches represents a core aspect of research into neuroadaptive technologies. These evaluations are often conducted under controlled laboratory settings and offline, where exhaustive analyses are possible. However, the manner in which classifiers are evaluated offline has been shown to impact reported accuracy levels, possibly biasing conclusions. In the current study, we investigated one of these sources of bias, the choice of cross-validation scheme, which is often not reported in sufficient detail. Across three independent electroencephalography (EEG) n-back datasets and 74 participants, we show how metrics and conclusions based on the same data can diverge with different cross-validation choices. A comparison of cross-validation schemes in which train and test subset boundaries either respect the block-structure of the data collection or not, illustrated how the relative performance of classifiers varies significantly with the evaluation method used. By computing bootstrapped 95% confidence intervals of differences across datasets, we showed that classification accuracies of Riemannian minimum distance (RMDM) classifiers may differ by up to 12.7% while those of a Filter Bank Common Spatial Pattern (FBCSP) based linear discriminant analysis (LDA) may differ by up to 30.4%. These differences across cross-validation implementations may impact the conclusions presented in research papers, which can complicate efforts to foster reproducibility. Our results exemplify why detailed reporting on data splitting procedures should become common practice.

## 1 Introduction

Neuroadaptive and passive Brain-Computer Interfaces (pBCIs) aim to improve human-machine interactions by providing the machine with neurophysiological information about the user. Using this information to estimate cognitive states like high mental workload and adapting the ongoing task accordingly could facilitate the development of personalized human-machine interactions (Fairclough, 2022; Zander and Kothe, 2011). A major challenge for this nascent type of technology is the development of neurophysiological-based classifiers that generalize across recording sessions, tasks, and people (Krusienski et al., 2011; Lotte et al., 2018; Saha and Baumert, 2020). These challenges are primarily driven by the non-linearities and non-stationarities inherent in electroencephalography (EEG), electrocorticography (EcoG), or magnetoencephalography (MEG) data, which are highly subject- and context-dependent (Krauledat, 2008; Mayer-Kress, 1998). However, these non-stationarities may also inflate pBCI model evaluation metrics, but this is sometimes overlooked in studies. Such is the case when model evaluation procedures fail to account for temporal dependencies between data used for training and testing classification models (Brouwer et al., 2015; Lemm et al., 2011). Positively biased evaluation metrics that essentially inform future research (such as choices regarding preprocessing, feature selection, and classification techniques) counteract the desirable goal of increasing reproducibility within pBCI research.

In the field of mental state classification, a considerable portion of research focuses on the development of new methodologies and comparative analyses of existing approaches (Demirezen et al., 2024). While it would be ideal to evaluate these methods in applied settings (Aricò et al., 2018; Lotte et al., 2018), in practice, achieving such evaluations poses considerable challenges (Brouwer et al., 2015). As a result, most model evaluations are conducted offline, typically through cross-validation procedures (Lemm et al., 2011).

Cross-validation serves as a key technique in this context, as it maximizes the use of available data by repeatedly partitioning data into training and testing subsets to compute evaluation metrics. The choice of cross-validation method is crucial, as it directly impacts the bias and variance of the evaluation metrics. Reducing bias typically requires larger training splits, which enhance model accuracy, whereas minimizing variance often necessitates larger testing splits, offering more robust estimates of evaluation metrics across iterations (Lemm et al., 2011).

Despite the ubiquity of cross-validation in the field, its implementation often lacks transparency (Li et al., 2020). In a review conducted by Demirezen et al. (2024), 93% of studies reported the cross-validation method used, while only 25% provided specific details regarding their data-splitting procedures. This lack of clarity complicates efforts to improve reproducibility—an issue highlighted as critical by researchers in the field (Gramann et al., 2024; Putze et al., 2022). Insufficient documentation of cross-validation details can hinder the assessment of bias-variance trade-offs and, in some cases, obfuscate issues regarding temporal dependencies between train and test splits (Li et al., 2020). In some cases, classification may actually be driven by temporal dependencies rather than class differences (Brouwer et al., 2015; Ivucic et al., 2024; Lemm et al., 2011; Li et al., 2020; Lotte et al., 2018; Riascos et al., 2024; Varoquaux et al., 2017; White and Power, 2023).

**Figure 1:**
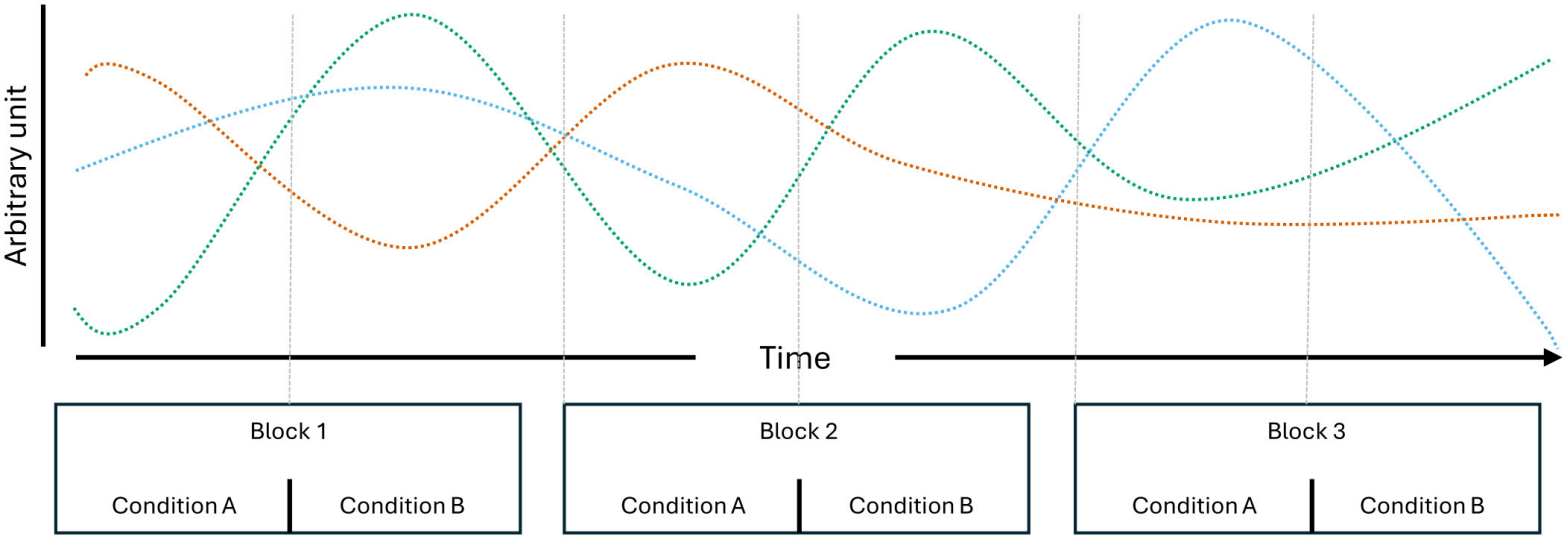
Schematic of multivariate temporal dependencies. Three individual processes, which, through their combinations, suffice to uniquely identify the individual condition repetitions presented over three blocks.

Temporal dependencies in neuroimaging data are likely to arise from various sources and exist across multiple timescales. Not only are they inherent to neural time-series (Bullmore et al., 2001; Linkenkaer-Hansen et al., 2001), but they may also be introduced due to experimental design choices. The recording hardware itself may be one source of such dependencies, such as when there are minor shifts or movements in the positions of EEG sensors. Other dependencies may stem from cognitive or behavioral factors. For instance, participants who start the session feeling nervous might gradually relax as they adapt to the experimental conditions. Increasing drowsiness may be visible in the theta and alpha (Strijkstra et al., 2003) as well as beta band of the EEG power spectrum (Aeschbach et al., 1997). Temporal dependencies may also present in more complex forms, as increasing drowsiness also affects theta- and alpha-specific connectivity metrics as well as the occurrence and prominence of microstates (Comsa et al., 2019). Initial nervousness, on the other hand, is often visible in heart rate dynamics (Lampert, 2015), which may affect the aperiodic activity (1/f slope) of the EEG power spectrum (Schmidt et al., 2024). Effects of bodily needs (i.e., hunger, thirst, dry eyes, caffeine/nicotine craving, etc.) may also exert influence after a certain point and affect EEG dynamics in unsuspecting ways. For example, when participants experience eye strain, they may start squinting their eyes, leading to power increases in higher frequency bands caused by the activations of facial muscles. In cases where data is split irrespective of the underlying block structure, the combination of a myriad of ongoing processes gives rise to multivariate temporal dependencies (Figure 1), which likely offer more information for classification than the class differences themselves (Ivucic et al., 2024; Li et al., 2020; Varoquaux et al., 2017; White and Power, 2023). The ways in which such temporal dependencies bias model evaluation metrics likely vary across cross-validation implementations, feature types, and classification algorithms, ultimately rendering conclusions drawn from underspecified cross-validation schemes unreliable sources of information.

The following section outlines previous work that explored the impact of different cross-validation schemes in neuroimaging research. The paper goes on to lay out what differentiates the current study from prior efforts.

Varoquaux et al. (2017) showed that leave-one-sample-out cross-validation schemes can inflate accuracy metrics due to temporal dependencies, overestimating performance across different fMRI decoding studies by up to 43% compared to evaluations on independent test sets. Model evaluation metrics from Leave-one-sample-out schemes are not only prone to bias from temporal dependencies but also suffer from high variance, due to the test set consisting of a single sample (Lemm et al., 2011; Varoquaux et al., 2017). K-fold cross-validation reduces the variance of model evaluation metrics by splitting the available sample data into k subsets, of which k-1 are used for training and the remaining subset is used to compute the evaluation metrics. This procedure is repeated k times until each subset was once used as a test set. However, temporal dependencies may also bias the results of k-fold cross-validation schemes when the available data is split into subsets without taking account of the underlying block/trial structure of the data. Ivucic et al. (2024) demonstrated that k-fold splits independent of trial structures caused inflated accuracy estimates in three open access EEG datasets dealing with auditory attention detection. Another inquiry demonstrated that deep-learning approaches for image and video classification based on EEG data fail entirely when stimuli are presented in a randomized rapid event-related fashion instead of a blocked design (Li et al., 2020).

With respect to pBCIs, one function of this technology is to differentiate between brain states (or cognitive/mental states), which may be considered more diffuse targets compared to the decoding of perceptual information. Manipulations aiming to induce specific emotional states or states of high mental workload may add systematic confounds that can bias model metrics (i.e., conditions differing in motor requirements; Brouwer et al., 2015). However, one major difference to domains like percept decoding or motor BCIs is that conditions tend to be presented in longer blocks of a single condition rather than short trials that allow for rapid event-based presentations with randomized condition orders. Interleaving 1 to 5-s-long trials of different motor conditions (e.g., left arm vs. right arm) or image conditions (e.g., houses vs. faces) assures that temporal dependencies are evenly spread across conditions. In the case of the Multi-Attribute Task Battery (MATB), a popular workload manipulation paradigm, a recent review has found duration of single condition presentations to range from 4–15 min (Pontiggia et al., 2024). Another popular paradigm for manipulating mental workload is the n-back, where block durations can be as short as 40 s (Shin et al., 2018) or as long as 10 min (Ke et al., 2021). Such long block durations increase the number of samples that share not just condition-specific dynamics but also the same temporal dependencies. Designing experiments with long blocks also reduces the number of repetitions of single conditions, which could be presented in a randomized order, in a standard-length recording session. Together, this range of factors complicates the evaluation of pBCIs in offline analyses.

White and Power (2023) focused on mental state classification and investigated the difference between block-independent k-fold splits and block-wise splits that assured samples from a single trial/block did not occur in both train and test subsets. Using open access EEG datasets manipulating emotional valence, they showed that k-fold accuracies were systematically higher than block-wise accuracies. They further showed that randomly reassigning class labels to half the blocks did not reduce the accuracy of the k-fold evaluations, concluding that the classifiers evaluated via k-fold made use of temporal dependencies rather than class differences. Further, they collected their own data, varying the trial durations of single condition repetitions (5, 15, and 60 s), thereby manipulating the number of samples sharing both class-labels and temporal dependencies. Here again, they demonstrated that a trial structure independent k-fold scheme overestimated accuracies, even using the short 15 s block durations. However, they also argue that their tested classifiers seemed to overfit on block-specific temporal dependencies when evaluated using the block-wise cross-validation scheme, leading to underestimated performance metrics (compared to extracting a single sample per block in the 5-s condition). While this study offered insight into how temporal dependencies can bias pBCI cross-validation results, their results may underestimate the issue, as their maximum trial length (60 s) was not reflecting the long block durations usually reported in pBCI experiments. Furthermore, all their tested classifiers used canonical band power features. However, the extent to which temporal dependencies bias cross-validation results may differ between the type of feature extracted from EEG, especially when additional dimensionality reduction techniques are applied during the training phase.

In the current study, we are focusing the inquiry into biased cross-validation metrics on three separate n-back datasets, as n-backs often serve as a workload manipulation paradigm that includes minimal motor-related confounds. We want to expand on previous works (Ivucic et al., 2024; Varoquaux et al., 2017; White and Power, 2023) by I exploring multiple classification pipelines and II adding additional cross-validation schemes. As cross-validation methods tend to ignore chronological order, they may underestimate the impact non-stationarities can have on model metrics (Riascos et al., 2024). Hence, we added a pseudo-online evaluation method in which only the very first occurrence of a condition is used for training. Lastly, we also added a worst-case scenario in which k-fold splits are not carried out on sequentially ordered data but rather on shuffled samples, which likely exacerbates the bias (Brouwer et al., 2015; Riascos et al., 2024).

## 2 Methods

To assess the impact of various cross-validation schemes on pBCI evaluation metrics independently of experimental design decisions, this analysis included three distinct datasets containing repeated presentations of three n-back conditions. Two were publicly available (Hinss et al., 2023; Shin et al., 2018) and one stems from our own previous study (Schroeder et al., 2024). These datasets differed in several aspects, including the n-back conditions employed, their presentation order, the intervals between repeated condition presentations, and the specifics of their EEG montage configurations. Following a general overview of the individual datasets, the methods for preprocessing and data selection to improve comparability are described.

### 2.1 Dataset descriptions

#### 2.1.1 Shin et al.

This study involved 26 participants completing 0-back, 2-back, and 3-back conditions. Numbers from 0–9 were used as stimuli, with targets making up 30% of the 20 trials that were presented per run. Each trial presented the stimulus for 0.5 s, followed by a 1.5 s fixation cross. Participants were instructed to respond to both targets and non-targets using a single hand (numpad 7 and 8 keys). Participants received instructions at the start of each run, but the authors provided no details about possible training periods before the data recording.

Conditions were presented in a counterbalanced order in blocks of 9 with 20 s breaks in-between runs. Three of these blocks were completed one after another before the experiment continued with different tasks not relevant to the current inquiry. It is unclear if participants took breaks between blocks, and if so, how much time passed between blocks.

EEG was recorded at 1,000 Hz with 30 active electrodes arranged according to the 10–5 system. Electrode TP9 was used as the online reference and TP8 as the ground. The authors did not report details about the impedance of the EEG electrodes.

#### 2.1.2 Hinss et al.

This study involved 29 participants completing 0-back, 1-back, and 2-back conditions. Numbers from 1–9 were used as stimuli, with targets making up 33% of the 48 trials that were presented per run. Each trial presented the stimulus for 0.5 s, followed by a 1.5 s fixation cross. Participants were instructed to only respond to targets using a single hand (spacebar).

Conditions were presented in a blocked format, each block containing 3 runs of a single condition without any reported times regarding breaks in-between runs. The blocks were randomly spread out over a 65–80 min-long recording period containing other tasks not relevant to the current inquiry, so the exact time between conditions is unknown. However, a special difference to the previous datasets is that participants returned twice (1 and 2 weeks after the first session) to repeat the experiment.

EEG was recorded at 500 Hz with 63 active electrodes arranged according to the 10–20 system. One electrode was sacrificed to record ECG. Electrode FCz was used as the online reference and Fpz as the ground. Electrode impedance was kept below 25 kOhm during the experiment.

#### 2.1.3 Schroeder et al.

This study involved 19 participants completing 1-back, 3-back, and 6-back conditions. A selection of letters (B, F, G, H, K, M, P, R, S, T, X, Z) was used as stimuli, with targets making up 30% of the 70 trials that were presented per run. Each trial presented the stimulus for 0.5 s followed by a 1.5 s question mark. Participants were instructed to respond to both targets and non-targets using both hands (Keyboard Z and M keys). Participants received extensive training (3 runs per condition) on a day prior to the experiment and practiced once more before recording data (1 run per condition).

Conditions were presented in blocks of 3, containing all 3 conditions in a randomized order. Exact data for how much time passed between runs within a block is not clear as participants were instructed to take self-paced breaks. In-between blocks, participants completed another task not relevant to the current inquiry, leading to at least 15 min separating the three blocks.

EEG was recorded at 500 Hz with 64 active electrodes arranged according to the 10–20 system. The Iz electrode was sacrificed and instead used as a peri-ocular EEG channel. Electrode FCz was used as the online reference and Fpz as the ground. Electrode impedance was kept below 25 kOhm during the experiment.

### 2.2 Dealing with the differences between datasets

**Figure 2:**
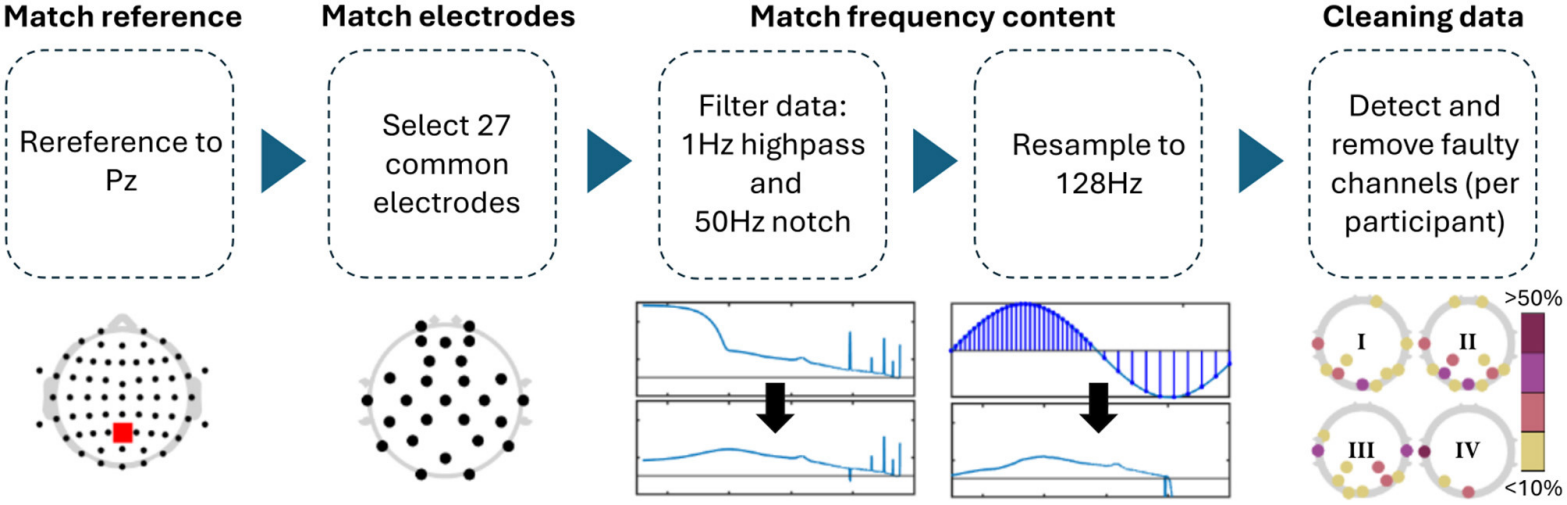
Preprocessing steps applied to all datasets. The topologies under the data cleaning step showcase that channel removal was not homogeneous across experiments, but mostly focused on posterior channels (I: single-day Hinss et al., II: multi-day Hinss et al., III: Shin et al., IV: Schroeder et al.).

Differences in the EEG recordings concerned the sampling frequency, online reference, number of channels used, and, to a lesser extent, the spacing of the electrodes. In order to make the EEG data more comparable across datasets, we preprocessed them to contain the same channel locations, reference electrode, and frequency content (Figure 2). Pre-processing was carried out in Matlab (Version: 2023a) and EEGLAB (v2021.1; Delorme and Makeig, 2004).

For the comparison, all channels in Schroeder et al. and Hinss et al. not contained in Shin et al. were removed. The only electrodes contained in Shin et al. that were not recorded in the other two datasets were AFF4 and AFF5. These are close in space to AF4 and AF5, which were retained in their stead with Hinss et al. and Schroeder et al. Since Cz was missing in the first 9 participants of Hinss et al., it was also removed from all three datasets. FCz was used as the online reference in Schroeder et al. and was recovered after computing a common average reference. For the comparison, all data was re-referenced to Pz, as this was one of only two shared midline electrodes between the datasets. Additionally, all data was notch filtered at 50 Hz (zero-phase, non-causal, with −6db cutoff frequencies at 49.25 and 50.75), highpass filtered at 1 Hz (zero-phase, non-causal, with −6dB cutoff frequency at 0.5 Hz), and resampled to 128 Hz to assure comparable frequency content across datasets. Lastly, EEGLAB's clean_rawdata function (v. 2.9.1) was used to remove channels when their correlation with neighboring channels was below 0.8 (see Table 1 for details).

**Table 1:** Channels removed per dataset.

**Dataset**	**Average # channels removed**	**Maximum # channels removed**
I. Single-day Hinss et al.	0.86	5
II. Multi-day Hinss et al.	1.69	6
III. Shin et al.	1.19	4
IV. Schroeder et al.	0.79	2

Average number of channels removed across participants and maximum number of channels removed for a single participant per dataset.

While the datasets listed above all made use of the n-back task, their n-back implementations likely differed in how demanding they were. The Schroeder et al. dataset placed the greatest working memory load on participants by utilizing a 6-back. However, the impossible nature of the 6-back may have led to effort withdrawal, rendering the condition less taxing than the dataset's 3-back. Both Hinss et al. and Shin et al. presented a 0-back, which removes the working memory aspect of the n-back and could be considered psychometrically distinct from other n-back conditions. For the comparison we decided to follow previous works described in the introduction and included two contrast per dataset—the widest difference in workload comparing the easiest and hardest conditions making for a higher class-separability contrast (Shin et al.: 0 vs. 3-back; Hinss et al.: 0 vs. 2-back; Schroeder et al.: 1 vs. 6-back) and the smallest difference in workload which we determined was the hardest and second hardest conditions making for a lower class-separability contrast (Shin et al.: 2 vs. 3-back; Hinss et al.: 1 vs. 2-back; Schroeder et al.: 3 vs. 6-back).

**Figure 3:**
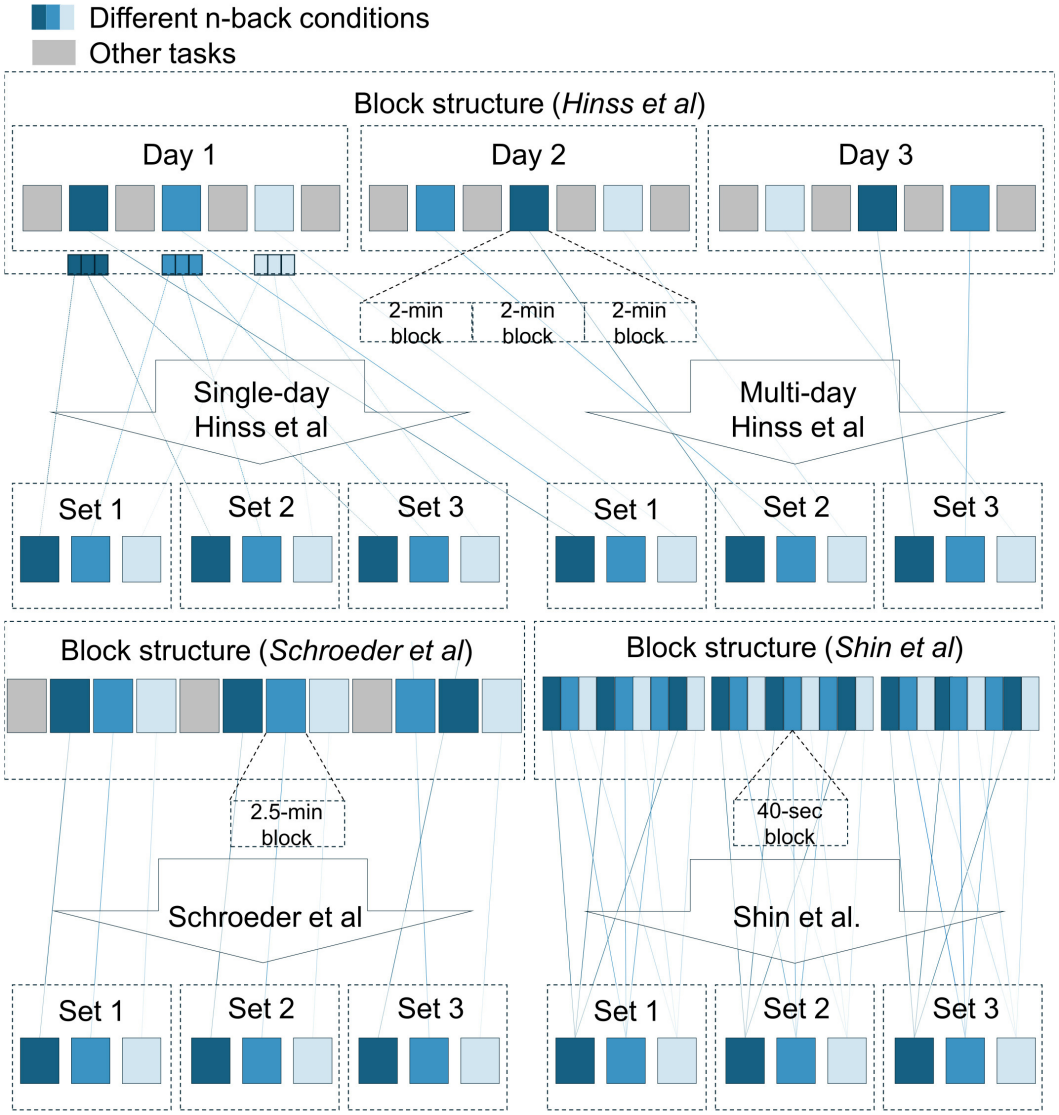
Block structure and splitting procedures for the three datasets. For each dataset, the available data was split into three equal-sized sets. Temporal dependencies were expected to be stronger within than between sets, as n-back blocks presented in conjunction with each other in the original experiments were grouped together within a single set. Presentation order of the n-back conditions was not necessarily as presented in the schematic.

Also important for the current inquiry was the order and spacing with which the n-back conditions were presented. Within a single session, the Hinss et al. dataset did randomize condition order, but repetitions of a single condition were grouped together in a single block. This likely resulted in temporal dependencies being highly informative to distinguish between conditions. However, the addition of two further recording days leaves this dataset with the most spread-out condition repetitions. Shin et al. and Schroeder et al. both only recorded on a single day but presented three spaced-out blocks of n-backs containing all three conditions in a pseudo-randomized order (Figure 3). Shin et al. placed these three blocks one after another, whereas Schroeder et al. spaced them out with other tasks in between, leaving at least 15 min between a repetition of a single condition. The blocks themselves were designed differently in Shin et al. and Schroeder et al. Schroeder et al.'s blocks contained one 140-s run per condition, whereas Shin et al. presented each condition three times in smaller 40-s runs concatenated together in a pseudo-random order, assuring a single condition was not presented twice in a row. Consequently, temporal dependencies are likely somewhat less informative for classification in the Shin et al. data compared to the other two datasets.

For the comparison, we arranged each participant's data into three sets (Figure 3). For Hinss et al., two separate procedures were adopted. The first only made use of the first recording day (96 s per condition per set) as an example of the additional bias introduced by the lack of a randomized and spaced-out presentation order. The second procedure made use of all three recording days. Each day was used as a separate set (288 s per condition per set). For Shin et al., runs of single condition (40 s each) within a block were sliced out of the continuous data and merged into a single file (120 s per condition per set). For Schroeder et al., the three condition repetitions were already spread out over the course of the experiment (140 s per condition per set). Samples within a set were expected to share more condition-unrelated information with each other, compared to samples from different sets, due to their temporal proximity in the original experiments (excluding the single-day Hinss et al. sets).

### 2.3 Classification approaches

All features were computed from 2-s windows, corresponding to the length of a single trial in the Shin et al. and Hinss et al. datasets. Windows were extracted without overlap from the continuous data, after classifier-specific filter operations were carried out. By extracting non-overlapping windows, we ensured that any bias to the performance metrics in the k-fold cross-validation schemes stemmed from underlying temporal dependencies and not from reusing the same data in successive samples. The classification approaches below were implemented using the pyRiemann (v0.7; Barachant et al., 2025), sci-kit learn (v1.2.2; Pedregosa et al., 2011) and MNE (v1.6.1; Gramfort et al., 2014) python libraries and are described in the order in which we expected them to overfit on training data specific temporal dependencies (likely showing greater bias in block-structure independent cross-validation strategies). This order was based on two ideas. The first being that with an increasing number of free parameters, the propensity of a classifier to overfit to training-specific information increases (Domingos, 2012; Lemm et al., 2011). The second being that Riemannian classification has previously been shown to generalize well to unseen data (Congedo et al., 2017; Yger et al., 2017).

#### 2.3.1 Broadband Riemann minimum distance to mean (RMDM)

Before windowing, the data for this classifier was bandpass filtered (1–25 Hz; default MNE FIR filter with −6 dB cutoff frequencies at 0.50 Hz and 28 Hz). Covariance matrices were computed per 2-s window with ledoit-wolf shrinkage (Ledoit and Wolf, 2004) to ensure semi-positive definite matrices. No hyperparameters were tuned, limiting the chance to overfit on trends in the training data. Classification was carried out using a Riemann minimum distance classifier (Barachant et al., 2010, 2012).

#### 2.3.2 Narrowband Riemann minimum distance to mean (narrow-RMDM)

Using a filter bank of butterworth filters (zero-phase, non-causal, passband ripple = 3db, stopband attenuation = 10 db, transition bandwidth = 1 Hz), the data for this classifier was separately bandpass filtered with passbands in canonical delta (1–4 Hz), theta (4–7 Hz), alpha (8–12 Hz), and beta (13–25 Hz) ranges. Ledoit-wolf shrinkage was carried out separately per frequency bin, after which all four frequency bins' covariance matrices were combined into a diagonal block matrix for classification. This procedure was inspired by the block-diagonal matrices proposed for the classification of SSVEPs (Congedo et al., 2017) and previous efforts to focus Riemannian classification on specific neurophysiological aspects (Näher et al., 2024; Yamamoto et al., 2023). To keep the computational requirements manageable, a previously proposed algorithm for efficient electrode selection on covariance matrices (Barachant and Bonnet, 2011) was used to reduce each frequency bin to its most informative combination of 8 electrodes at each training step. This pipeline was included as an example of a Riemannian classifier with an added train-set specific tuning step. Due to the fact that different channels could be selected per frequency band, all off-diagonal elements in the block-covariance matrix (theoretically containing cross-frequency coupling information) were set to 0.

#### 2.3.3 Narrowband power LDA (PSD-LDA)

After windowing, we computed the power spectral density within canonical delta, theta, alpha, and beta ranges per electrode using a one-dimensional discrete Fourier Transform. During training, all features were normalized using the mean and standard deviation of the training set. Additionally, every training iteration, the 18 most informative and least correlated features were selected from the 4 (frequency bands) x n_electrodes number of computed features using a minimum redundancy maximum relevancy algorithm (Peng et al., 2005).

#### 2.3.4 Filter bank common spatial pattern LDA (FBCSP)

Using a filter bank of butterworth filters (zero-phase, non-causal, passband ripple = 3 db, stopband attenuation = 10 db, transition bandwidth = 1 Hz), the data for this classifier was separately bandpass filtered into 4 Hz wide frequency bins ranging from 3–25 Hz in steps of 2 Hz. Each filtered signal was used to compute 8 spatial filters via common spatial pattern analysis (4 highest and 4 lowest eigenvalues) (Ang et al., 2012; implemented in MNE) and the log-variance of the filtered 2-s windows was extracted as the classification feature. Every training iteration, a subset of 18 features was selected from the 80 computed features (10 frequency bins x 8 spatial filters) using a minimum redundancy maximum relevancy algorithm to select the most informative and least correlated features (Peng et al., 2005).

### 2.4 Cross-validation strategies

**Figure 4:**
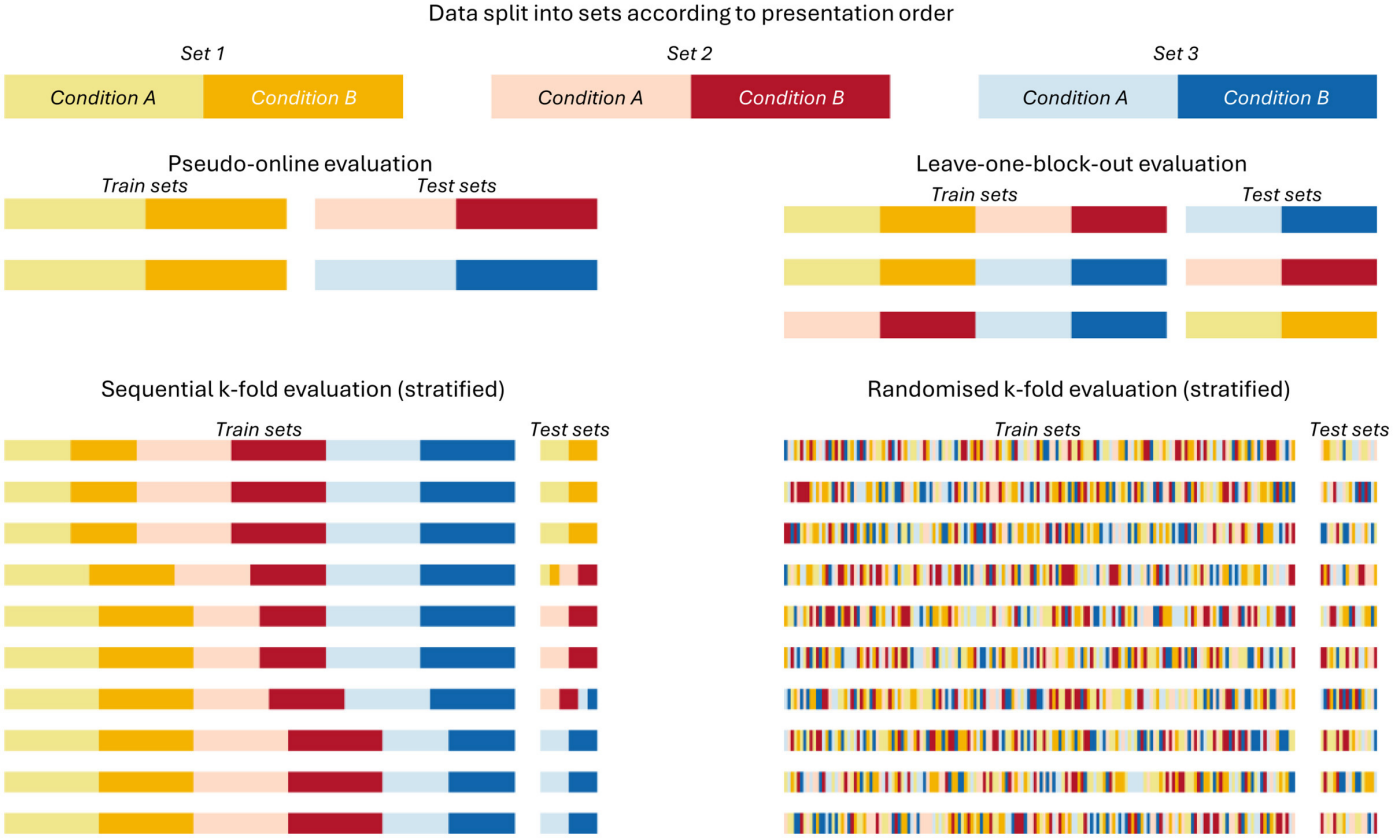
Splitting procedure of the tested cross-validation strategies. Visualization of how the separate sets built in Figure 3 were split for training and testing the classifiers within the four cross-validation procedures.

Four different validation strategies were tested (Figure 4), ranging from likely producing conservative performance estimates to likely producing inflated performance estimates and presented in that order below. We computed classification accuracy as our performance metric, as all cross-validation methods used assured no class imbalances in the train or test sets (see Supplementary Table 1 for exact sample sizes).

#### 2.4.1 Pseudo-online

The most conservative of the four tested cross-validation strategies. In it, only the first set of conditions was used for training and the remaining two sets were used for testing. This evaluation scheme represents situations in which limited calibration data is available, such a “cold-starting” a new model for a user in an applied setting. The single training set mimics a calibration round while the test sets offer two separate opportunities to estimate classifier performance without making use of future data, as is the case in the following cross-validation strategies.

#### 2.4.2 Leave-one-block-out

This strategy assured that no data of the same set occurred in both the testing and training data. However, using this strategy, some folds will use training data that occurred after the testing data. Since this is impossible for a real BCI system, its performance estimates could be considered somewhat artificial and might be overestimated (Riascos et al., 2024).

#### 2.4.3 Sequential K-fold

This is a common default cross-validation strategy in which the data are split into, here, 10 equal-sized segments. 9 of the 10 segments are used for training, while the 10th is held out for testing. This process is repeated 10 times until all samples were once used for testing. The risk of splitting the data into equal sized segments of an arbitrary size is that data from a single session may occur both in the training and testing sets within a single fold. We used a stratified k-fold procedure to avoid class imbalances.

#### 2.4.4 Randomized K-fold

Here as well, the data were split into 10 equal-sized segments, using a stratified procedure to avoid class imbalances. However, in this version, all samples are first shuffled randomly before being split into 10 folds, a step that should only be performed if all observations are statistically independent from each other. We included it here as an example of the worst-case scenario for overestimating classifier performance on data from block-based experiments.

### 2.5 Statistical analysis

**Figure 5:**
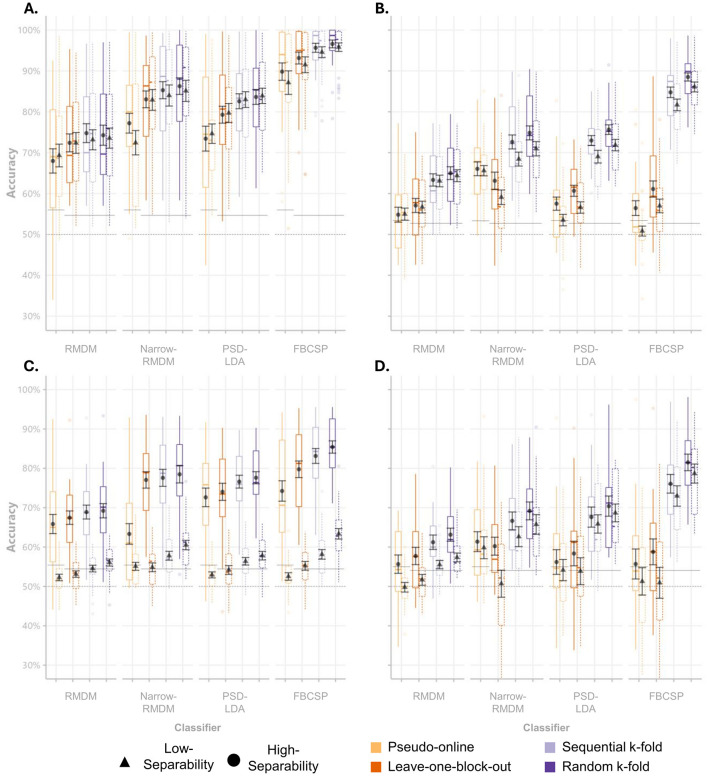
Accuracy scores per classifier and cross-validation scheme. Boxplots of mean classification accuracies for single-day Hinss et al. **(A)**, multi-day Hinss et al. **(B)**, Shin et al. **(C)**, and Schroeder et al. **(D)**. Black point ranges represent the mean accuracies across subjects and their standard errors. The dashed horizontal lines display the theoretical chance level, while the solid horizontal lines display the average sample size corrected chance levels.

As a first step, the distributions and descriptive statistics of subject-wise classification accuracies were visualized for each dataset, cross-validation strategy, and classification approach (see Figure 5).

To analyse differences between cross-validation strategies, we computed bootstrapped 95% confidence intervals of the differences in accuracy between the conservative pseudo-online cross-validation scheme and the other tested schemes over 10.000 iterations per classifier. Each iteration randomly sampled 15 subjects with replacement across datasets and class-separability contrasts to compute the 3 difference scores per classifier (within-subject).

We further analyzed the impact of cross-validation schemes on comparisons between classifiers across datasets. To investigate this, we tested for significant differences among the four classifiers within the low and high class-separability contrasts for each cross-validation scheme. Non-parametric Friedman tests were employed to account for the non-normality of the data. Additionally, *post-hoc* Durbin-Conover pairwise comparisons were conducted to provide more detailed insights into whether the differences between classifiers varied across evaluation schemes. *P*-values of the pairwise comparisons were adjusted using the Benjamini-Hochberg procedure to control the false discovery rate (FDR) at α = 0.05 (using the PMCRplus package—Pohlert, 2024).

## 3 Results

Across all datasets, we could observe the expected increases going from pseudo-online to the randomized k-fold cross-validation scheme (Figure 5). The most noticeable inflation from conservative to the block-structure independent schemes was visible for the FBCSP classifier. Interestingly, differences between the high and low-separability contrasts seem to be maintained even in the inflated accuracy estimates of the two k-fold approaches (e.g., if we see a difference between high and low separability in the conservative schemes, it is also visible in the biased schemes). A Friedman test across datasets, classifiers and cross-validation strategies [χ^2^(1) = 25.252, *p* < 0.001], followed by pairwise Wilcoxon signed-rank tests conducted per dataset, revealed that only the data from Shin et al. exhibited significant differences in the classification accuracy between the low and high class-separability contrasts across cross-validation schemes and classifiers (*p* < 0.001).

Looking at the two panels belonging to the Hinss et al. dataset, we can observe the effect of not interleaving conditions with each other. Using only the first day of their dataset, in which three repetitions of a single condition were presented in sequence, we observed on average 10.1% higher accuracy estimates compared to the panel next to it (which used data from all 3 days), even for the conservative pseudo-online and leave-one-block out cross-validation schemes.

### 3.1 Impact of cross validation choices across datasets

To get a general idea of how different cross-validation strategies affected estimates of model accuracy, Table 2 displays the bootstrapped mean classification accuracies across datasets with additional bootstrapped 95% confidence intervals for each cross-validation strategy's difference to the most conservative strategy (pseudo-online). The confidence intervals were computed across datasets and class-separability contrasts, excluding the *single-day* Hinss et al. dataset because the lack of randomization for that dataset portrayed a different violation of independence as described in section 2.2.

**Table 2:** Bootstrapped average accuracies and differences by cross-validation scheme.

**Classifier**	**Pseudo-online**	**Leave-one-block-out**	**Sequential K-fold**	**Randomized K-fold**
Broad-RMDM	59.4% (–)	61.7% (−1%, 4.4%)	65% (2.5%, 9%)	66% (3.6%, 10.7%)
Narrow-RMDM	65.7% (–)	67.4% (−5.8%, 4%)	72.7% (1.67%, 10.2%)	74.6% (3.8%, 12.7%)
PSD-LDA	62.5% (–)	65.4% (−0.8%, 4.3%)	72.4% (6.6%, 14.6%)	73.3% (8.4%, 17.1%)
FBCSP	65.9% (–)	69.8% (−0.1%, 7.9%)	81.8% (13.3%, 26%)	85.2% (17.5%, 30.4%)

Average classification accuracies across class-separability contrasts and experiments (excluding single-day Hinss et al. results) with bootstrapped 95% confidence intervals of the differences between each cross-validation scheme and the pseudo-online results.

### 3.2 Pair-wise comparisons across datasets

**Figure 6:**
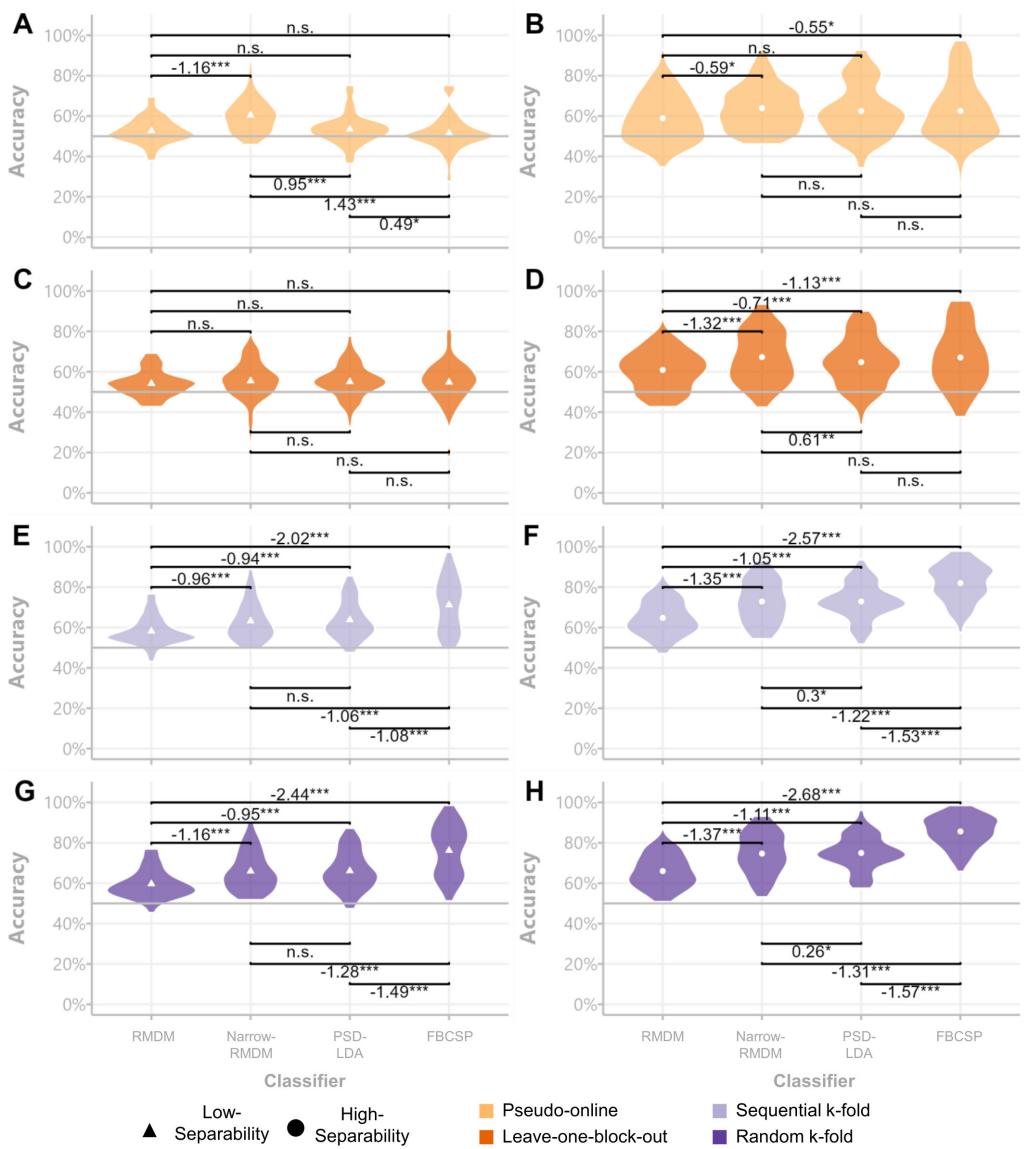
Classifier comparisons across datasets and cross-validation schemes. Violin plots of subject-wise accuracy scores per class separability contrasts. Low-separability results are displayed in the left column **(A, C, E, G)** and high-separability results in the right column **(B, D, F, H)**. Significance was assessed via Durbin-Conover pair-wise comparisons and average rank differences (left minus right) are displayed next to the significance signifiers. *p < 0.05, **p < 0.01, ***p < 0.001.

The Friedman tests conducted on the pseudo-online evaluations revealed significant effects for both the low class-separability contrast [χ^2^(3) = 51.867, *p* ≤ 0.001] and the high class-separability contrast [χ^2^(3) = 9.795, *p* ≤ 0.001]. In the low class-separability context, pair-wise comparisons showed the Narrow-RMDM classifier outperformed all other classification approaches (see Figure 6A). In the high class-separability context, the PSD-LDA, Narrow-RMDM, and FBCSP approaches did not differ significantly from each other (Figure 6B). For the leave-one-block-out evaluation scheme, the low class-separability contrast did not show significant differences between classifiers [χ^2^(3) = 5.475, *p* = 0.14]. However, significant differences were observed for the high class-separability contrast [χ^2^(3) = 45.944, *p* ≤ 0.001], with pair-wise comparisons now showing the FBCSP classifier to be significantly more accurate than the broadband RMDM and PSD-LDA classifiers. The performance differences between the Narrow-RMDM and FBCSP classifiers were no longer significant (see Figure 6D).

In the sequential k-fold evaluation, both the low [χ^2^(3) = 91.276, *p* ≤ 0.001) and high class-separability contrasts [χ^2^(3) = 150.74, *p* ≤ 0.001] displayed significant differences. In this case, the FBCSP classifier demonstrated significantly better performance compared to the Narrow-RMDM classifier (see Figures 6E, F). This performance difference remained highly significant in the randomized k-fold evaluation, where significant differences were again observed for both the low [χ^2^(3) = 135.05, *p* ≤ 0.001) and high [χ^2^(3) = 162.84, *p* ≤ 0.001] class-separability contrasts.

## 4 Discussion

The objective of the current study was to investigate the extent to which pBCI model evaluation metrics may be biased when temporal dependencies between train and test samples are not considered in cross-validation. To achieve this, we used data from three datasets involving the n-back task, which often serves as a workload manipulation paradigm with minimal motor-related confounds. Our analysis evaluated four classifiers, ranging from models using Riemannian minimum distance metrics on minimally pre-processed broadband EEG data to models using supervised dimensionality reduction on narrowband filtered EEG data. The results showed that not observing temporal dependencies in cross-validation methods impacts model evaluation metrics to a great degree, as reported in other areas dealing with neuroimaging-based classification (Ivucic et al., 2024; Li et al., 2020; Varoquaux et al., 2017; White and Power, 2023). Importantly, our results additionally showed that this bias is not equal across classifiers. Models with greater propensity to tune to the available training data (Domingos, 2012; Lemm et al., 2011) tended to outperform alternative models in block-structure independent cross-validation schemes, while they performed at similar levels or even significantly less accurately in more conservative evaluation schemes. Consequently, model comparisons based on offline cross-validation need to be interpreted carefully and should be questioned if cross-validation splits were carried out independently of the experiment's block structure or not thoroughly documented.

The most conservative validation method assessed in this study, pseudo-online evaluation, adhered strictly to the chronological order of the data and utilized only a single data block for model training. This reflects scenarios such as calibrating a classifier for a new user in real-time classification. In our implementation, the pseudo-online evaluation likely provided overly conservative performance estimates. This was by design, as we were interested in comparing the other schemes to a lower bound. It did indeed result in the lowest classification accuracies (Table 2), frequently failing to surpass adjusted chance levels in all datasets (Figure 5). In contrast, the leave-one-block-out cross-validation scheme, which also preserved the block structure but disregarded the temporal order of samples, achieved better-than-chance classification across a greater number of classifiers by training on two data blocks instead of one. Notably, none of the four tested classification approaches showed significantly higher accuracy comparing the leave-one-block-out evaluation to the pseudo-online evaluation (all bootstrapped 95% CIs in Table 2 included 0), even though their training data was double in size.

When comparing the conservative evaluation methods to the two k-fold cross-validation approaches, where data splitting disregarded the experimental block structure, significant inflations in classification accuracy were observed across all classifiers we tested. For the simple RMDM approach, accuracy increased by up to 9%, while the electrode-selection variant of RMDM displayed increases of up to 12.7% accuracy. Even more pronounced increases were evident in the two LDA classifiers, which utilized canonical band power features combined with additional dimensionality reduction techniques. When only employing feature selection on band power features during the training phase, accuracy estimates rose by up to 17.1% compared to the pseudo-online evaluation. Adding another dimensionality reduction step (FBCSP) further inflated accuracy estimates, increasing them by up to 30.4% (Table 2).

Against expectations, the upper bounds of the confidence intervals for the sequential and random k-fold approaches did not differ substantially across the three datasets tested (Table 2). This was surprising, as the random k-fold approach represents a more obvious violation of the assumption of independence. The observation that merely sharing non-overlapping data of a single session in train and test sets can cause similar biases in the accuracy metrics, demonstrates the issue of temporal dependencies in offline pBCI model evaluations aptly. An additional analysis demonstrating that condition-related similarity and similarity due to temporal proximity are conflated in block-based experiments can be found in the online Supplementary material 2.

Although the four evaluation schemes varied in the sizes of their training sets, these variations alone do not suffice to explain the results presented. The leave-one-block-out cross-validation, despite two times the training data compared to the pseudo-online evaluation, showed negligible accuracy gains. In contrast, k-fold methods differed much more from the pseudo-online evaluation with a not quite threefold increase in training set size.

Furthermore, since the degree of inflation to model accuracy seemed to differ between feature/classifier types, model comparisons based on evaluation metrics computed on block-structure independent cross-validation schemes may lead to erroneous conclusions that would not replicate in applied settings. We demonstrated this in section 3.2 where the FBCSP classifier showed great advantages over the other tested classifiers in the k-fold evaluations, but was either not significantly different to its alternatives in the high-separability case and was actually outperformed by the Narrow-RMDM in the low-class separability case (Figure 6). In general, it is to be expected that the propensity to overfit on training-specific information increases with classifier complexity (Domingos, 2012; Lemm et al., 2011)—a problem underlined by the extreme differences found between evaluation schemes used for deep-learning-based M/EEG decoding (Ivucic et al., 2024; Li et al., 2020).

Interestingly, of the tested high-separability contrasts, the Shin et al. dataset showed the best performance in the pseudo-online cross-validation scheme (Figure 5). The difference to the other datasets might be linked to the manner in which conditions were interleaved with each other within a single presentation block in the Shin et al. dataset. Interleaving several shorter repetitions of different conditions likely caused different classes to share the same temporal trends, leaving the trends less informative for the classification task. Another contributing factor could have been that the high separability contrast contained the 0-back condition. Reacting to a single stimulus in a stream rather than having to repeatedly encode and maintain new stimuli could be considered psychometrically distinct from the other n-back conditions, making for an easier classification problem (Gerjets et al., 2014).

We also included an example of only making use of a single day of the Hinss et al. dataset (see Figure 5A; not included in the other analyses). Here, all blocks of a single condition were presented in sequence together, leading to samples of a single class sharing temporal dependencies regardless of whether they stemmed from a single or separate blocks. Due to the lack of randomization, classifiers were likely utilizing temporal dependencies regardless of the cross-validation scheme used. Results garnered using the data from a single day were, on average, 10.1% higher than those from the full multi-day dataset. Using the Pseudo-online or leave-one-block-out cross-validation scheme on a single day (rather than all 3 days) would erroneously lead to the conclusion that the FBCSP classifier performed the best of the four tested classifiers (i.e. the classifier with the highest propensity to utilize temporal dependencies instead of class differences).

The various differences between the methods of the three datasets described in 2.1 could be viewed as a hindrance to our cross-dataset analyses. We considered them a strength, as the reported results appeared consistent regardless of the differences pertaining to participant training regime, presentation order, condition contrast, etc. Similar results could likely be garnered from various kinds of block-based experimental designs with (pseudo-) randomized condition orders. However, as demonstrated through the example of only using a single day of the Hinss et al. data, the results may differ if conditions were presented without randomization. Furthermore, slower physiological signals, such as fNIRS or ECG, may exhibit even stronger biases in block-structure independent cross-validation due to their slowly evolving nature, which may be accompanied by longer-lasting temporal dependencies (Blanco et al., 2024). Finally, as alluded to in the introduction, the bias stemming from splitting training and testing data irrespective of experimental structure can be avoided in event-based experimental designs, provided that the condition order is fully randomized and no more than a single sample is drawn from any given trial (White and Power, 2023).

The results we presented should by no means be viewed as a complete treatment of the issue at hand. Future studies could further deepen the understanding of biases in pBCI model evaluation by focusing on specific facets in pBCI processing pipelines. For the sake of parsimony, the current study did not include data cleaning steps beyond the removal of faulty channels. Employing additional cleaning steps like artifact subspace reconstruction (ASR) or removing artifacts via independent component analysis (ICA) would likely improve the accuracy metrics we reported here (Liu et al., 2021; Sannelli et al., 2009). However, their implementation details, as well as the length and selection of calibration data, may produce different dynamics across cross-validation methods and should be explored in a dedicated inquiry. We also avoided tuning hyperparameters to individual subjects to simplify the current analyses. Given our results, the addition of nested cross-validation to tune various hyperparameters would likely exacerbate the influence of temporal dependencies in block structure independent evaluation schemes. Lastly, the offline evaluation of adaptive machine learning approaches may require their own detailed investigation into similar evaluation biases, as their methods aim to actively remedy mismatches between data distributions used for training and testing (Kumar et al., 2019; Lotte et al., 2018; Schlögl et al., 2010).

### 4.1 Key takeaways

- To avoid the biased model evaluation discussed in the current study, experimental manipulations should ideally be delivered on a trial-by-trial basis to allow for better randomization and reduce the likelihood of conflating condition differences with temporal dependencies. Importantly, if train and test sets consist of shuffled samples, only a single sample should be extracted per trial (White and Power, 2023).
- If blocked designs are required, data splitting for model evaluation should consider the experimental structure and be documented in great detail to increase transparency for the sake of reproducibility. Given the superior pseudo-online classification results in the Shin et al. dataset, it may also be favorable to opt for many shorter blocks rather than fewer longer blocks.
- Model comparisons based on insufficiently documented data splitting procedures should be interpreted with caution, as complex models are likely more prone to overfit on training-specific trends in block-structure-independent evaluation schemes, leading to more pronounced performance metric inflation compared to their less complex counterparts.

## 5 Conclusion

Our study adds to the literature exploring the impact that different choices in cross-validation practices can have on pBCI evaluation metrics across three sets of n-back data. While the problem of temporal dependencies in neuroimaging data is well known (Lemm et al., 2011; Linkenkaer-Hansen et al., 2001; Bullmore et al., 2001), the prevalence of underreported details on cross-validation methods (Demirezen et al., 2024; Li et al., 2020) prompted us to revisit the issue in this current study. The bias introduced by not observing temporal dependencies when splitting EEG data for cross-validation favors models with more training data-specific optimisation steps, possibly inviting false conclusions about their true generalizability. This should motivate researchers to ask themselves whether they have provided enough information in a given study to assure colleagues and stakeholders that they did everything in their power to limit the influence of temporal dependencies before drawing conclusions from their model evaluation metrics.

## Data Availability

The data analyzed in this study is subject to the following licenses/restrictions: One of the three used datasets is currently not open access. It will only be made publicly available upon approval of the funding institute after completion of the project. Requests to access these datasets should be directed to sch.felix.sch@gmail.com.
