# Supplementary material for: The impact of cross-validation choices on pBCI classification metrics: lessons for transparent reporting
Source: Front Neuroergon. 2025 Jul 1;6:1582724. doi: 10.3389/fnrgo.2025.1582724 (PMC12259573; doi:10.3389/fnrgo.2025.1582724)
Supplement: Supplementary file 1 [file Supplementary_file_1.docx]

Supplementary Table

# Average sample sizes used for training and testing classifiers

**Supplementary Table 1.**

| CV-strategy | Dataset | Condition A training samples | Condition B training samples | Condition A testing samples | Condition B testing samples |
| --- | --- | --- | --- | --- | --- |
| Pseudo-online | Single-day Hinss et al | 51 | 51 | 98 | 98 |
|  | Multi-day Hinss et al | 158 | 158 | 295 | 295 |
|  | Schroeder et al | 70 | 70 | 140 | 140 |
|  | Shin et al | 60 | 120 | 60 | 120 |
| Leave-one-block-out | Single-day Hinss et al | 102 | 102 | 49 | 49 |
|  | Multi-day Hinss et al | 310 | 310 | 149 | 149 |
|  | Schroeder et al | 140 | 140 | 70 | 70 |
|  | Shin et al | 120 | 120 | 60 | 60 |
| Sequential k-fold | Single-day Hinss et al | 138 | 138 | 14 | 14 |
|  | Multi-day Hinss et al | 418 | 418 | 44 | 44 |
|  | Schroeder et al | 189 | 189 | 21 | 21 |
|  | Shin et al | 162 | 162 | 18 | 18 |
| Randomsied k-fold | Single-day Hinss et al | 138 | 138 | 14 | 14 |
|  | Multi-day Hinss et al | 418 | 418 | 44 | 44 |
|  | Schroeder et al | 189 | 189 | 21 | 21 |
|  | Shin et al | 162 | 162 | 18 | 18 |

Note. The average sample size per cross-validation fold available for training and testing the four classifiers.
