# Supplementary material for: The impact of cross-validation choices on pBCI classification metrics: lessons for transparent reporting
Source: Front Neuroergon. 2025 Jul 1;6:1582724. doi: 10.3389/fnrgo.2025.1582724 (PMC12259573; doi:10.3389/fnrgo.2025.1582724)
Supplement: Supplementary file 2 [file Supplementary_file_2.docx]

Supplementary Analysis

# Testing for Multicollinearity between condition and latency-based predictors

We utilised a procedure inspired by representational similarity analysis (RSA) to clarify how temporal dependencies may be conflated with condition differences. By showing how much variance in the covariance structure of EEG data can be explained by the temporal proximity of two samples, we hope to elucidate the problem of block-structure independent train and test splits from a different perspective.

First, continuous data were segmented into non-overlapping 2-second windows, using the same preprocessing used for the main analysis. Each window was associated with its corresponding onset latency (seconds from experiment start) and experimental condition label (i.e., the dataset-specific n-back conditions).

After computing ledoit-wolf regularised covariance matrices per window, we calculated all pairwise Riemann distances per participant. This resulted in a symmetric pairwise time-by-time dissimilarity matrix for each participant, where larger distances indicated greater dissimilarity in neural activity patterns.

We then constructed two participant-specific model-based predictors:

1. **Condition Model:** A theoretical matrix encoding the relationship between trial conditions. Pairs of windows from the same condition were assigned a value of 0, pairs spanning adjacent difficulty levels (easy-medium or medium-hard) were assigned 0.5, and pairs spanning the largest difficulty gap (easy-hard) were assigned 1. This model reflects expected dissimilarity based on task difficulty.
2. **Latency Model:** A temporal proximity model based on a Gaussian decay function. For two windows with latencies t1​ and t2​, the model assigned similarity as:

$$S\left( t1,t2 \right)=1 - e^{-\frac{\left( t1-t2 \right)^{2}}{{2\sigma}^{2}}}$$

where σ = 30 seconds. This value was chosen to allow for a relatively slow decay, so similarity decreased significantly only after ~60 seconds (2σ). This model captures the hypothesis that temporally adjacent samples are more similar, irrespective of task condition.

We extracted and vectorised the upper triangular portion from each participant’s dissimilarity matrix and the two model matrices (excluding the diagonal). These vectors were then rank-transformed in accordance with previous recommendations (Kriegeskorte et al., 2008) before fitting the different models.

To quantify the contribution of each model, we performed the following regression analyses on ranks per participant:

- **Model 1:**

$$Y=\beta_{0}+\beta_{1}X_{Condition}+\epsilon$$

where Y is the rank-transformed dissimilarity vector, and $X_{Condition}$ is the rank-transformed condition model vector. This regression estimates the variance in dissimilarity explained by task condition alone.

- **Model 2:**

$$Y=\beta_{0}+\beta_{1}X_{Latency}+\epsilon$$

where Y is the rank-transformed dissimilarity vector, and $X_{Latency}$ is the rank-transformed latency model vector. This regression estimates the variance in dissimilarity explained by temporal proximity.

- **Model 3 (Residual Analysis):**

We computed residuals from Model 2 to isolate variance not explained by latency:

$$Yr=Y-\hat{Y}_{Latency}$$

Then, we regressed these residuals on the condition model:

$$Yr=\beta_{0}+\beta_{1}X_{Condition}+\epsilon$$

This step estimated the unique contribution of the condition after accounting for the variance, which could also be explained by temporal proximity.

Finally, we tested whether the variance accounted for by temporal proximity (at σ = 30) in the ranked data overlapped significantly with the variance accounted for by condition information. We did this by running a paired t-test per dataset on the Fisher z-transformed R^2^ values of the participant-wise fits of the condition model and the partial fits of the condition model after removing the variance explained by the latency model. The results showed a significant overlap for the *Schroeder et al* (t(18) = 3.15, p < 0.01 - Supplementary Figure 1B) and *Hinss et al* datasets (t(28) = 4.5, p < 0.01 - Supplementary Figure 1C). The effect was non-significant for the *Shin et al* data (t(18) = 3.15, p < 0.01 - Supplementary Figure 1A)

The non-significant result in *Shin et al* may be due to their shorter block durations. When the latency model explains variance due to temporal proximity beyond 60 seconds and the individual blocks only lasted 40 seconds, the residuals after fitting the latency model may actually remove condition-unrelated information, thereby suppressing noise and improving the fit of the condition model in some cases. For the *Hinss et al* and *Schroeder et al* data, similar dynamics could may also be observed at larger sigma values.

**Model fitting results per dataset**


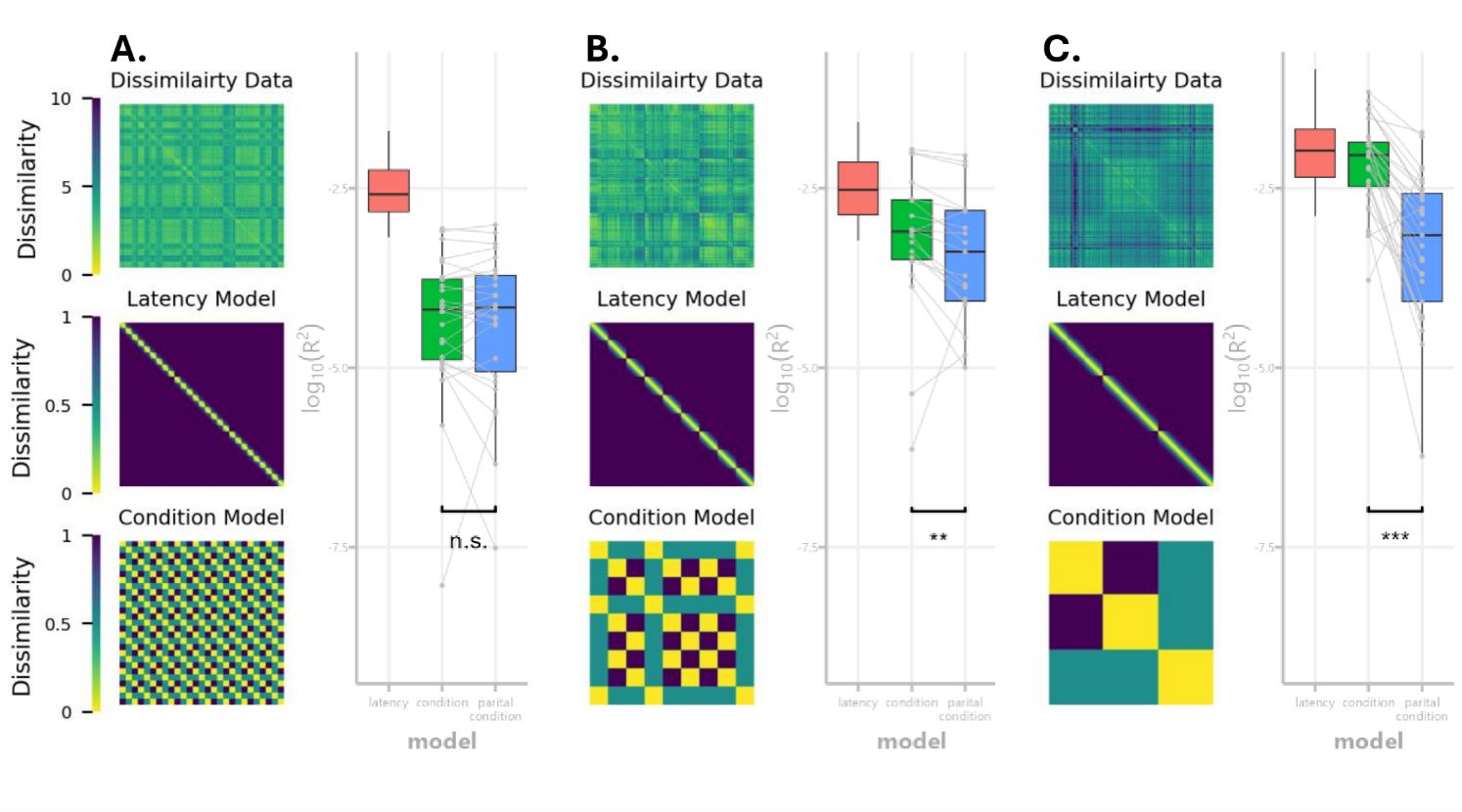


**Supplementary Figure 1.** Model fitting results and exemplary empirical, latency model, and condition model time-by-time dissimilarity matrices per dataset. A. presents the results for *Shin et al*. B. presents the results for the *Schroeder et al* data. C. presents the results for the first day of the *Hinss et al* data. p < .05 = *, p < .01 = **, p < .001 = ***, n.s. = non-significant.

# References

Kriegeskorte, N., Mur, M., & Bandettini, P. A. (2008). Representational similarity analysis—Connecting the branches of systems neuroscience. Frontiers in Systems Neuroscience, 2. https://doi.org/10.3389/neuro.06.004.2008
